# Aplastic anemia following high-voltage electrical injury: A case report

**DOI:** 10.1016/j.tcr.2025.101258

**Published:** 2025-10-06

**Authors:** Mehdi Ayaz, Dorsa Ayaz, Asma Keshavarz

**Affiliations:** a Shiraz University of Medical Sciences, Iran; b Isfahan University of Medical Sciences, Iran

**Keywords:** High-voltage electrical injury, Aplastic anemia, Pancytopenia, Burn complications, Bone marrow suppression, Hematological complication, Case report, Transient aplastic anemia, Electrical burn, Rare complication

## Abstract

**Introduction:**

High-voltage electrical injuries (HVEI) can lead to a wide range of complications, including musculoskeletal, neurological, cardiac, and renal damage. Hematological complications are rare but have been reported, with aplastic anemia being an exceptionally uncommon consequence. To date, only two cases of aplastic anemia following HVEI have been documented.

**Case presentation:**

We report the case of a 21-year-old male who sustained a HVEI and developed progressive pancytopenia during hospitalization. Despite stable vital signs and initially normal lab results, his hemoglobin dropped significantly by day 17, followed by a marked decline in white blood cell and platelet counts. Bone marrow biopsy confirmed the diagnosis of aplastic anemia. The patient had no prior hematological conditions or evidence of infection, and his medications were not known to cause bone marrow suppression. He was treated with supportive care, G-CSF, corticosteroids, and Danazol. After several weeks, his blood counts gradually recovered, and full remission was confirmed one month post-discharge.

**Discussion:**

This case highlights the potential for bone marrow suppression and aplastic anemia following HVEI, even in the absence of infection or drug-induced toxicity. The delayed onset of pancytopenia suggests a need for ongoing hematological monitoring in similar patients. This case contributes to the limited literature on this rare complication and emphasizes the importance of early recognition and management.

**Conclusion:**

Aplastic anemia is a rare but serious complication of HVEI. Clinicians should maintain a high index of suspicion for hematological abnormalities in burn patients, particularly in the weeks following injury. Early diagnosis and treatment are essential for favorable outcomes.

## Introduction

Electrical injuries are responsible for approximately 1000 fatalities and 30,000 non-fatal shock incidents annually in the United States. They also rank as the fourth leading cause of occupational trauma-related deaths [1]. Electrical injuries are classified as low voltage (<1000 V), high voltage (>1000 V), and lightning injury. High voltage current results in more severe injuries and is more likely to cause internal damage [2]. These injuries can result in a wide range of complications, including musculoskeletal, behavioral, neurological, cardiac, and renal issues [3,4]. While hematological complications are rare, they have been documented and include conditions such as thromboembolism [[4], [5], [6]] and disseminated intravascular coagulation (DIC) [7].

One particularly rare hematological complication, which is the focus of this paper, has been previously reported only twice: once in 2020 in a 30-month-old Iranian boy [8] and again in 1984 in a 19-year-old male after an electrical injury [9]. While it is challenging to definitively link this case to the electrical injury, we believe that reporting this rare occurrence can raise awareness among clinicians. This may facilitate better management and early detection of such complications in future cases.

## Case presentation

A 21-year-old male sustained a high-voltage electrical injury (HVEI) while working with electrical equipment. After initial resuscitation at a local hospital, he was transferred to the “Ghotbodin” Burn Center in Shiraz, Fars province, southern Iran. Upon arrival, his vital signs were stable:

• Temperature: 37.7 °C.
• Blood Pressure: 120/75 mmHg.
• Pulse: 108/min.
• Respiratory rate: 22/min.

Physical examination revealed **4th-degree full-thickness burns** involving approximately **10** **% of the total body surface area (TBSA)**, specifically affecting the **face, right hand, and both feet**. There were no signs of infection or other trauma, and the rest of the exam was normal.

**Fig. 1:**
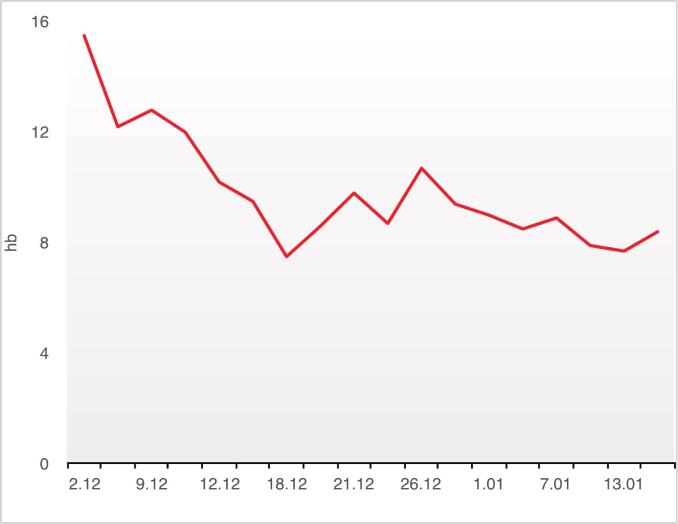
Trend of changes in the patient's hemoglobin (Hb) levels during the hospital course.

**Fig. 2:**
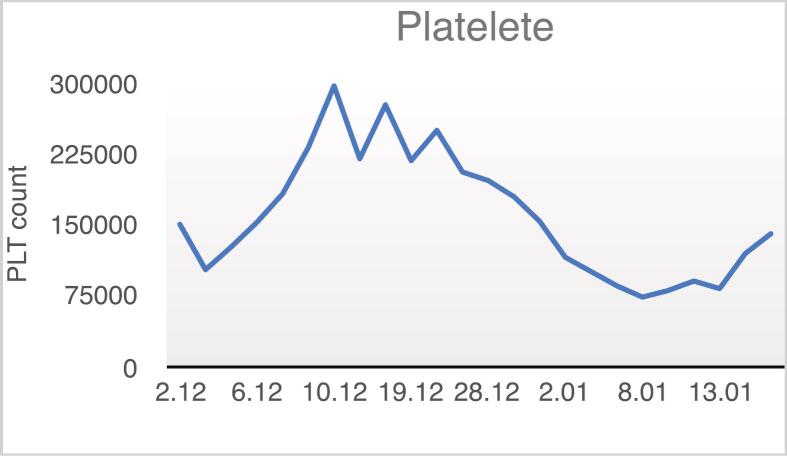
Trend of changes in the patient's platelet (Plt) count over time.

**Fig. 3:**
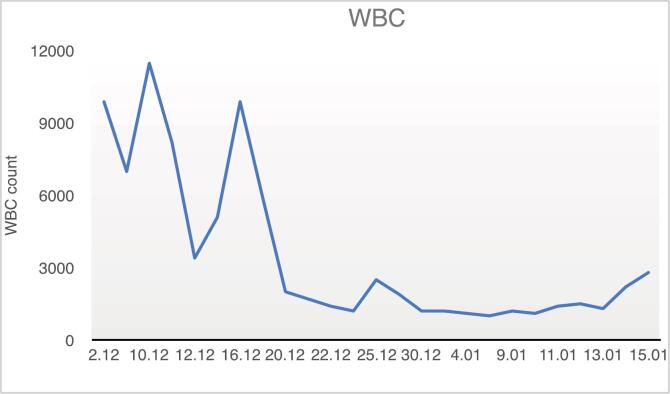
Trend of changes in the patient's white blood cell (WBC) count during the hospital course.

He underwent two surgeries for debridement, excision, and grafting of his deep burn wounds with minimal blood loss (10th and 16th December 2012). Regular laboratory tests, including a complete blood count (CBC), were all normal until day 17, when dramatic changes in his WBC and hemoglobin levels were observed (Fig. 1, Fig. 2, Fig. 3).

On day 17, the patient's hemoglobin dropped to 7.5 g/dL, and six days later, his WBC count fell to 1200 cells/μL. To address this, we started granulocyte colony-stimulating factor (G-CSF), administered when WBC count dropped below 2000 cells/μL. By day 33, his platelet count also dropped with no improvement in WBC or hemoglobin. Because G-CSF wasn't effective, the patient received 4 units of packed red blood cells (RBC) and 22 bags of fresh frozen plasma (FFP).

The patient had no history of hematological disorders, and no medications appeared to be responsible for his condition. Physical examination showed no signs of infection or bleeding, except for the burn wounds. Hematology specialists were consulted, and they ordered body fluid cultures (blood, wound tissue, and urine), all of which came back negative. They recommended starting Danazol and Prednisolone and conducting further tests, including a peripheral blood smear (PBS) and bone marrow biopsy.

**Fig. 4:**
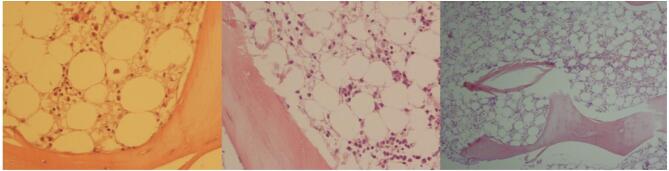
Bone marrow biopsy showing increased adipose tissue with scattered lymphoplasmacytic cells. Hematopoietic cells are severely decreased. (H&E × 100).

The PBS showed hypochromic microcytic anemia, severe leukopenia, and granulocytopenia, with a mild decrease in platelet count. A bone marrow biopsy revealed markedly reduced hematopoietic cells, which led to a diagnosis of aplastic anemia (Fig. 4).

After 46 days in the hospital, the patient was discharged with blood counts that had not yet returned to normal (WBC: 2800 cells/μL, Hb: 8.4 g/dL, RBC: 3,100,000 cells/μL, Hct: 25.9 %, Plt: 140,000 cells/μL). Throughout his treatment, he received 22 bags of FFP, six bags of packed RBC, and several doses of G-CSF.

When he returned for another visit one month later, his blood counts had fully recovered (WBC: 4200 cells/μL, Hb: 14.8 g/dL, Hct: 46 %, Plt: 133,000 cells/μL).

## Discussion

Prolonged hospitalization in patients with electrical burns is primarily due to the unique characteristics of these injuries. Although less frequent than other types of burns, electrical burns often cause deeper and more extensive tissue destruction [10]. This variation in injury mechanism and tissue involvement makes electrical burns more complex and time-intensive to treat, even when the affected total body surface area (TBSA) is comparable to burns from other causes.

Electrical burns are categorized based on voltage level—low voltage (<1000 V), high voltage (>1000 V), and lightning-related injuries. Among these, high-voltage injuries tend to cause more significant internal damage [2]. In the case presented, the patient experienced a high-voltage electrical burn.

The trajectory of the electrical current through the body determines which organs and tissues are impacted. Typically, electricity enters through the hand and exits through the foot [10]. In this case, the current entered through the patient's right hand and exited through the left foot.

Approximately three weeks after the incident, the patient exhibited transient bone marrow suppression.

When an electrical current traverses the body, it can disrupt normal cellular functions [2]. Several mechanisms have been proposed to explain the resulting tissue damage, but the most widely accepted involves the transformation of electrical energy into heat. While nerves and blood vessels conduct electricity efficiently, bones, skin, and fat are more resistant, generating heat in the process. This thermal effect can cause deep tissue injury—particularly to muscle overlying bones—while leaving superficial tissues less affected [16]. We suggest that bone marrow may also be susceptible to damage through this thermal mechanism. When the electrical current is intense, or traverses a large segment of the body, or if the patient has limited bone marrow reserves, the result may be a reduction in blood cell counts. Among the various blood cells, those with highly sensitive precursors in the bone marrow are particularly at risk.

In this patient, laboratory findings suggested a diagnosis of transient aplastic anemia (AA), a condition marked by pancytopenia and reduced bone marrow cellularity.

While many cases of AA are classified as idiopathic, secondary causes—such as infections, medications, or malignancies—should also be considered. Historically, instances of bone marrow suppression post-burn injury have been primarily linked to infections [12]. However, our patient had no signs of active infection, and all cultures remained negative. Furthermore, the medications he was prescribed during his hospital stay are not commonly associated with bone marrow suppression. The antibiotics administered included Ceftazidime (2 days), Vancomycin (29 days), Imipenem (8 days), Amikacin (5 days), Piperacillin (16 days), and Meropenem (12 days), given either prophylactically or based on clinical suspicion. These antibiotics were discontinued once negative blood cultures were confirmed (before initiating systemic antibiotic therapy). None of these agents are known to cause bone marrow suppression in vivo [11].

Although rare complications such as leukopenia from Meropenem or thrombocytopenia from Vancomycin have been reported, these do not account for our patient's pancytopenia, as all three major blood cell lines were affected [13,14].

Diagnosis of aplastic anemia is usually based on the presence of pancytopenia alongside a hypocellular bone marrow with fatty infiltration. Although pancytopenia is the typical presentation, early stages can sometimes involve the depression of only one or two blood cell lines, which may later evolve into full pancytopenia [15]. In our case, the blood cell decline began with anemia, was followed by leukopenia, and ultimately progressed to thrombocytopenia.

Although aplastic anemia following electrical injuries is rare, this case adds to the two previously reported instances: one in a 30-month-old Iranian boy in 2020 [7] and another in a 19-year-old male in 1984 [8]. Given these reports, clinicians need to consider the possibility of hematological complications, including aplastic anemia, in patients who have experienced high-voltage electrical injuries, particularly if hematological parameters suddenly deteriorate after the initial recovery phase. This case highlights the necessity of vigilant, ongoing monitoring of blood counts in such patients, as complications like pancytopenia can develop weeks post-injury.

Early recognition of these complications is crucial for ensuring appropriate intervention and minimizing further morbidity. While this complication remains rare, its recurrence in multiple cases suggests that it is a potential risk that should not be overlooked in the management of electrical burn patients.

## Conclusion

This case highlights the rare occurrence of aplastic anemia following high-voltage electrical injury. Although uncommon, hematological complications such as pancytopenia should be considered in the weeks following such injuries. Early recognition and intervention are crucial to prevent further complications. This case, along with previous reports, underscores the importance of monitoring hematological parameters in electrical burn patients.

## CRediT authorship contribution statement

**Mehdi Ayaz:** Conceptualization, Resources, Writing – original draft, Project administration, Validation, Methodology, Supervision, Writing – review & editing. **Dorsa Ayaz:** Writing – review & editing. **Asma Keshavarz:** Writing – original draft.

## Patient consent

Written informed consent was obtained from the patient for the publication of this case report, including any accompanying images.

## Images and illustrations

The images and illustrations presented in this report are original and have been used with the patient's consent.

## Declaration of Generative Al and Al-assisted technologies in the writing process

This case report utilized ChatGPT solely for language correction and manuscript editing. No AI tools were used in the diagnosis, treatment, or clinical management of the patient. The authors take full responsibility for the content, analysis, and conclusions presented in this case report.

## Funding

The authors declare that no funding was received for this case report.

## Declaration of competing interest

The authors declare that they have no known competing financial interests or personal relationships that could have appeared to influence the work reported in this paper.
